# Protective effect of purple sweet potatoes (*Ipomoea batatas* L.) against rat breast cancer

**DOI:** 10.14202/vetworld.2025.1137-1146

**Published:** 2025-05-13

**Authors:** Carmen R. Silva-Correa, Julio Hilario-Vargas, Abhel A. Calderón-Peña, Víctor E. Villarreal-La Torre, Cinthya L. Aspajo-Villalaz, Natalia Bailon-Moscoso, Juan Carlos Romero-Benavides, Oscar Herrera-Calderon, William A. Sagástegui-Guarniz, Julio A. Castañeda-Carranza, Walter E. Janampa-Castillo, José L. Cruzado-Razco

**Affiliations:** 1 Department of Pharmacology, Faculty of Pharmacy and Biochemistry, National University of Trujillo, Trujillo, Perú; 2 Department of Human Physiology, Faculty of Medicine, National University of Trujillo, Trujillo, Perú; 3 Department of Biological Chemistry and Animal Physiology, Faculty of Biological Sciences, National University of Trujillo, Trujillo, Perú; 4 Departamento de Ciencias de la Salud, Facultad de Ciencias de la Salud, Universidad Técnica Particular de Loja, Loja, Ecuador; 5 Departamento de Química, Facultad de Ciencias Exactas y Naturales, Universidad Técnica Particular de Loja, Loja, Ecuador; 6 Department of Pharmacology, Bromatology and Toxicology, Faculty of Pharmacy and Biochemistry, Universidad Nacional Mayor de San Marcos, Jr Puno 1002, Lima 15001, Peru; 7 Department of Statistics, Faculty of Physical and Mathematical Sciences, National University of Trujillo, Trujillo, Perú; 8 Department of Psychological Sciences, Faculty of Education and Communication Sciences, National University of Trujillo, Trujillo, Perú

**Keywords:** 7,12-dimethylbenz(a)anthracene, anthocyanins, antioxidant activity, breast cancer, *Ipomoea batatas*, tumor latency

## Abstract

**Background and Aim:**

Breast cancer is one of the most prevalent and lethal malignancies affecting women worldwide. Given the limitations of conventional treatments, there is an increasing interest in exploring naturally derived compounds with chemoprotective properties. Purple sweet potatoes (*Ipomoea batatas* L.) are rich in anthocyanins and have been reported to possess antioxidant, anti-inflammatory, and anticancer activities. This study aimed to evaluate the chemopreventive potential of ethanolic extracts from purple sweet potato peels in a rat model of 7,12-dimethylbenz(a)anthracene (DMBA)-induced breast cancer.

**Materials and Methods:**

Fifty female *Rattus norvegicus* (170–200 g) were randomized into five groups. Breast tumors were induced through a single subcutaneous dose of DMBA (20 mg/rat). Three experimental groups received daily oral administration of the extract at 200, 400, and 600 mg/kg body weight, respectively, for 4 months. One control group received only DMBA, while another received the highest dose of the extract without DMBA. Antioxidant activity was assessed using 2,2-diphenyl-1-picrylhydrazyl-hydrate (DPPH) and 2,2’-Azino-bis(3-ethylbenzothiazoline-6-sulfonic acid) assays. Anthocyanin content was quantified using spectrophotometry. Tumor latency, tumor volume, and histopathological alterations were evaluated to determine the extract’s chemopreventive effects.

**Results:**

The extract exhibited significant antioxidant activity comparable to quercetin at 1500 ppm (DPPH assay) and a high anthocyanin content (138.92 ± 0.58 mg/100 g dry extract). Tumor latency was significantly prolonged in the 600 mg/kg group (101 days) compared to the DMBA control (88 days). In addition, this group showed a marked reduction in tumor volume (2.26 cm^3^ vs. 15.21 cm^3^; p < 0.05). Histological examination revealed improved ductal epithelial integrity and reduced necrosis in extract-treated groups, particularly at the highest dose.

**Conclusion:**

The ethanolic extract of purple sweet potato peels demonstrated a dose-dependent chemopreventive effect against DMBA-induced breast cancer in rats. The extract’s high anthocyanin content likely contributed to its antioxidant and antitumor activities. These findings suggest potential applications in dietary chemoprevention, warranting further investigation into its molecular mechanisms and clinical translation.

## INTRODUCTION

Cancer is defined by the unregulated proliferation and division of cells, which can result in tumor development or metastasis – the process by which cancer cells invade and spread to distant parts of the body [1]. Metastasis remains the leading cause of morbidity and mortality among cancer patients [2]. Breast cancer is the most frequently diagnosed carcinoma in women and represents the primary cause of cancer-related mortality in numerous countries [3]. Globally, the majority of breast cancers are classified as either ductal or lobular subtypes, with ductal carcinoma accounting for approximately 40%–75% of all diagnosed cases [4]. Despite notable advances in oncology [5], conventional treatment modalities continue to face substantial challenges [6, 7], including adverse toxicity profiles and the emergence of therapeutic resistance [8, 9].

This study investigates the potential of purple sweet potato peels, which are rich in anthocyanins, as a safer, naturally derived alternative for breast cancer prevention. *Ipomoea batatas* L. exhibits various ecotypes, distinguished by the differing coloration of its root peel and pulp [10]; this coloration is associated with health-promoting properties. For example, deeper yellow pigmentation indicates a higher concentration of carotenoids, primarily β-carotene. Yellow- and orange-fleshed sweet potatoes are rich in phenolic acids, whereas purple varieties contain markedly elevated levels of anthocyanins [11], including nonacylated and acylated forms of peonidin, cyanidin, and pelargonidin glycosides [12]. Consequently, purple-fleshed sweet potatoes possess greater quantities of phenolic acids and flavonoids than the other two types [13]. The phytochemical composition of *I. batatas* underpins its wide range of medicinal properties, including antioxidant, immunomodulatory [14], anti-inflammatory [15], hypoglycemic [16], cardioprotective, antimicrobial, and anticancer activities [17].

Anthocyanin-rich extracts derived from *I. batatas* have demonstrated inhibitory effects against various cancer cell lines, such as Michigan Cancer Foundation-7 (MCF-7) (breast cancer), Seoul National University-1 (gastric cancer), and derivative of another colon adenocarcinoma cell line (colon adenocarcinoma) [18]. *In vivo* studies using murine models of induced colorectal cancer have also confirmed its anticancer potential [19]. Other investigations have focused on polysaccharides from *I. batatas*, which protect macrophages from lipopolysaccharide-induced damage by exhibiting anti-inflammatory effects and suppressing elevated phagocytosis, as well as tumor necrosis factor-alpha and interleukin-6 levels [20]. In addition, glycoproteins such as sweet potato proteoglycans (SPG-8700 and SPG-56), isolated from purple varieties of *I. batatas*, have been implicated in anticancer activities against colon and breast cancer cell lines [21, 22]. Phytosterols extracted from *I. batatas*, including daucosterol linolenate, daucosterol linoleate, and daucosterol palmitate, have been shown to inhibit the growth of MCF-7 breast cancer cells and reduce tumor progression in 7,12-dimethylbenz(a)anthracene (DMBA)-induced murine breast cancer models [23].

Despite significant advances in cancer thera-peutics, breast cancer remains a leading cause of morbidity and mortality among women worldwide. Conventional treatments such as chemotherapy, radiation, and hormone therapy are often associated with adverse effects and the development of drug resistance [6–9]. Consequently, there is an urgent need to identify alternative, safer therapeutic agents, particu-larly from natural sources with fewer side effects. While *I. batatas* (purple sweet potato) has been extensively studied for its antioxidant, anti-inflammatory, and antimicrobial properties, its chemopreventive potential against breast cancer, especially using peel extracts rich in anthocyanins, remains underexplored. Existing literature has primarily focused on the anticancer effects of anthocyanins in colon and gastric cancers, with limited *in vivo* evidence supporting their efficacy against mammary carcinogenesis. Moreover, few studies have investigated the dose-dependent effects of anthocyanin-rich extracts in animal models of chemically induced breast cancer, particularly in relation to tumor latency, volume, and histopathological outcomes.

This study aimed to evaluate the chemopreventive effects of an ethanolic extract of purple sweet potato *(I. batatas* L.) peels in a rat model of DMBA-induced breast cancer. Specifically, it sought to assess the antioxidant capacity, total anthocyanin content (TAC), and dose-dependent effects of the extract on tumor latency, tumor volume, and histopathological alterations in mammary tissue. By elucidating the potential protective role of *I. batatas* peel extract, this study contributes novel insights into the development of functional foods or nutraceuticals as complementary strategies in breast cancer prevention and therapy.

## MATERIALS AND METHODS

### Ethical approval

The animal study protocol was approved by the Ethics Committee of the Faculty of Medicine at the Universidad Nacional de Trujillo (Approval Certificate No.: 014 - 2021/UNT-FM-C.E.).

### Study period and location

The study was conducted from January 2022 to July 2023. All procedures were carried out in the Toxicology Laboratory, School of Pharmacy and Biochemistry, Universidad Nacional de Trujillo, Perú.

### Chemicals and reagents

DMBA was procured from Sigma-Aldrich Co. (St. Louis, MO, USA). Neutral-buffered formalin (10%), hematoxylin, eosin, and hydrochloric acid fuming (37%) were supplied by Merck® (Darmstadt, Germany). Ethyl alcohol (96°) was obtained from Alkofarma E.I.R.L. (Lima, Perú). Sodium pentobarbital Halatal® (6.5%) and sodium chloride (0.9%) were provided by Medifarma (Lima, Perú).

### Plant material and extraction

The purple sweet potato (*I. batatas*) was collected from the town center of La Constancia, District of Simbal, La Libertad Region, Perú. A voucher specimen was deposited in the Herbarium Truxillensis (HUT) of the National University of Trujillo under the code 61442. For extraction, 5 kg of sweet potato peel, previously chopped into small pieces, was macerated for 72 h in 96° ethyl alcohol acidified to pH 3.5 using 1.5 N hydrochloric acid. The resulting extract was filtered and oven-dried at 45°C for 48 h to yield a dry extract, which was subsequently stored frozen at −20°C [24].

### Determination of antioxidant activity

#### 2,2-diphenyl-1-picrylhydrazyl-hydrate (DPPH) assay

The antioxidant activity of the extract was evaluated using the DPPH scavenging capacity assay. The assay was conducted in a 96-well microplate by adding 20 μL of the extract at various concentrations (1.95, 3.9, 7.81, 15.62, 31.25, 62.5, 125, 250, 500, 1000, 1500, and 2000 ppm) to 180 μL of a 0.1 mM DPPH solution. After incubation in the dark for 30 min at room temperature (23°C), the absorbance was measured at 517 nm using a microplate reader. Methanol was used as the blank. The scavenging effect (%) was calculated using the following formula:


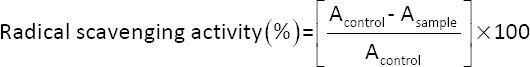


Ascorbic acid and quercetin were used as positive standards. All tests were performed in triplicate [25].

#### 2,2’-Azino-bis(3-ethylbenzothiazoline-6-sulfonic acid) (ABTS) assay

The ABTS reagent was prepared by mixing 5 mL of 7 mM ABTS with 88 μL of 140 mM potassium persulfate. This mixture was incubated in the dark at room temperature for 16 h to generate free radicals and then diluted with distilled water at a ratio of 1:44 (v/v). For the determination of scavenging activity, 100 μL of the ABTS reagent was mixed with 100 μL of the sample in a 96-well microplate and incubated at room temperature for 6 min. After incubation, absorbance was measured at 734 nm using a microplate reader, with methanol used as a blank. The scavenging effect (%) was calculated using the same formula described in the DPPH assay.

Ascorbic acid and quercetin were used as positive controls. All tests were conducted in triplicate [26].

### Quantification of TAC

The TAC was quantified according to the standardized protocol of Giusti and Wrolstad [27]. To extract anthocyanins, 0.5 g of the dry extract was placed in 15 mL Falcon tubes, and 10 mL of 96% ethanol acidified to pH 2 with 1 N HCl was added. The mixture was homogenized using a Corning vortex for 1 min and centrifuged for 10 min at 4000 rpm in a Labfish centrifuge (model LC-8S). The supernatant (S1) was collected. Subsequent re-extractions were performed on the remaining residue using 6 mL of acidified ethanol for each step, vortexed for 1 min, and centrifuged again. The supernatants from these extractions (S2 and S3) were collected and combined with S1 in a 25 mL volumetric flask, and the volume was adjusted with acidified ethanol.

To determine TAC, two 1:2 dilutions of the extract were prepared – one in acetate buffer (pH 4.5) and the other in HCl/KCl buffer (pH 1). These were allowed to stand in the dark for 15 min. The absorbance of each dilution was measured at 515 nm and 700 nm using a Spectrophotometer (Jenway™, England, 7305 Model), against a blank of double-distilled water. All measurements were performed in triplicate using the following expression:


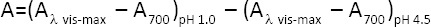


Where:

A_λ vis-max_ is the maximum absorbance at pH 1.0 and pH 4.5 and A_700_ is the absorbance at 700 nm at pH 1.0 and pH 4.5.

TAC (in mg/L) was calculated using the following formula:


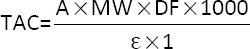


Where:

TAC: Total anthocyanin content; A: Absorbance of the sample calculated according to the previous equation; DF: dilution factor (2); MW: Molecular weight of cyanidin-3-glucoside = 449.6 g/mol; ε: Molar absorptivity of cyanidin-3-glucoside = 26 900 L/mol/cm.

### Evaluation of anticancer activity

#### Animals

Fifty female *Rattus norvegicus* var. *albinus*, aged 50–60 days and weighing between 170 and 200 g, were obtained from the Animal Center of the School of Pharmacy and Biochemistry. The animals were housed under standard conditions and provided with a balanced diet and water *ad libitum*.

#### Breast cancer induction

Breast cancer was induced by subcutaneous administration of DMBA, diluted in 1 mL of olive oil, at a single dose of 20 mg into the mammary tissue [28]. The animals were randomly divided into five groups, each consisting of ten female rats. Group I (DMBA Control) received a single dose of DMBA and was treated with physiological saline for 4 months. Groups II, III, and IV received DMBA (single dose) followed by daily oral administration of the ethanolic extract of *I. batatas* at doses of 200, 400, and 600 mg/kg/day, respectively, for 4 months. Group V (Extract Control) received 600 mg/kg/day of *I. batatas* extract without DMBA exposure. All treatments continued for 4 months.

#### Evaluation of tumor reduction

Weekly palpation was conducted to monitor tumor development in the thoracoabdominal and inguinal regions, facilitating the assessment of tumor latency. At the end of the experiment, the animals were euthanized through an overdose of pentobarbital, and the tumors were excised for volume measurement. Tumor dimensions (height, length, and width) were measured using a Vernier caliper [29].

The accumulated tumor volume was calculated using the following formula:


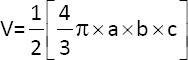


Where V = accumulated tumor volume; a = width; b = length; c = height.

#### Histopathological analysis of tumors

Each animal was dissected using previously sterilized instruments. The excised tumors were preserved in 10% buffered formalin and submitted for histopathological examination. Tissue sections were stained with hematoxylin and eosin, and the slides were examined under an optical microscope (Motic, Spain) to evaluate morphological alterations [29].

### Statistical analysis

Tumor latency and tumor volume data were analyzed using R software, licensed by the GNU Project (Version 4.3.1) for Windows® (https://www.r-project.org/). Data were subjected to analysis of variance, followed by Tukey’s *post hoc* test for pairwise comparisons. Statistical significance was considered at p < 0.05.

## RESULTS

### Determination of antioxidant activity

The antioxidant activity of the purple sweet potato extract was assessed using DPPH and ABTS assays and compared with that of ascorbic acid and quercetin across a range of concentrations (1.95–2000 ppm). In the DPPH assay, the extract demonstrated antioxidant capacity comparable to that of ascorbic acid and quercetin at a concentration of 1500 ppm (p < 0.05). However, in the ABTS assay, the antioxidant capacities of quercetin and ascorbic acid were significantly higher than that of the sweet potato extract.

### Quantification of TAC

The results revealed a high TAC in the ethanolic extract of purple sweet potato (*I. batatas*) peels, amounting to 138.92 ± 0.58 mg of anthocyanins per 100 g of dry extract, quantified as cyanidin-3-glucoside.

### Evaluation of anticancer activity

**Figure 1:**
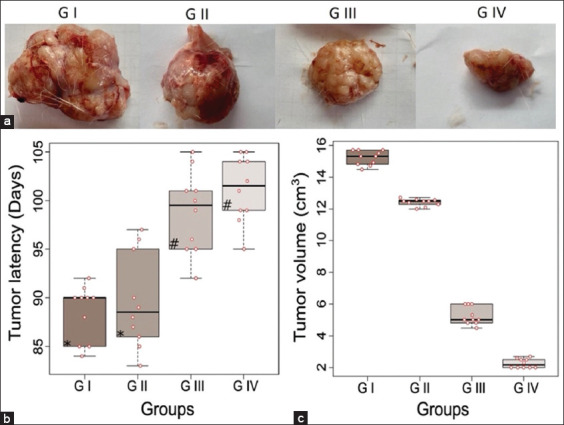
Effects of purple sweet potatoes (*Ipomoea batatas*) on (a) tumor formation, (b) latency, and (c) volume in rats with 7,12-dimethylbenz(a)anthracene -induced breast cancer. All data are presented as mean ± standard deviation (n = 10 per group, p < 0.05, analysis of variance, *post hoc* Tukey honestly significant difference test. * and ^#^indicate statistical similarity between groups.

Figure 1 illustrates the various parameters analyzed to evaluate the anticancer activity of *I. batatas* in rats with DMBA-induced breast cancer. Specifically, Figure 1a displays tumor formation in each experimental group, Figure 1b shows tumor latency (time to appearance), and Figure 1c presents tumor volume. Significant differences were observed among the experimental groups (p < 0.05), particularly in Group IV, which received 600 mg/kg of the *I. batatas* extract and exhibited the longest latency period (101 days), compared to the DMBA control group (88 days) (Figure 1b). A marked reduction in tumor volume was also evident in the treatment groups, with Group IV showing the most substantial decrease (2.26 cm^3^) compared to the control group (15.21 cm^3^) (p < 0.05) (Figure 1c).

### Histopathological analysis of tumors

**Figure 2:**
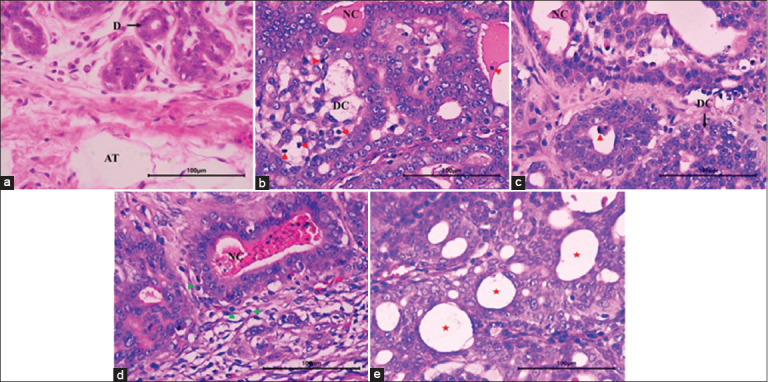
Photomicrographs of histological changes in breast tissue (hematoxylin and eosin, 40×). (a) Group I: control 7,12-dimethylbenz(a)anthracene, (b) Group II: *Ipomoea batatas*-200 mg/kg/día, (c) Group III: *I. batatas*-400 mg/kg/día, (d) Group IV: *I. batatas*-600 mg/kg/day, and (e) Group V: control *I. batatas: I. batatas*-600 mg/kg/day. DC=Ductal carcinoma *in situ*, NC=Necrosis, red arrowhead: microcalcifications, CT-D=Dense connective tissue; green arrowhead: lymphocyte, Red star=Cystic fibrous changes with cystically dilated irregular ducts, AT=Adipose tissue, D=Ducts.

Figure 2 presents the histological alterations observed in breast tissues across the experimental groups. In Group I (Control–DMBA), histological features characteristic of ductal carcinoma *in situ* were evident, including prominent microcalcifications and marked necrosis. Group II exhibited ductal carcinoma *in situ* with localized necrosis and inflammatory infiltration. In Group III, necrotic areas and intravascular lymphatic infiltration were observed; however, the restoration of the ductal epithelium remained limited. In contrast, Group IV demonstrated partial regeneration, with areas of ductal hyperplasia and notable recovery of the ductal epithelial structure, alongside cystic and fibrous changes. Finally, Group V (Control–*I. batatas*) displayed normal histological architecture, comprising inactive breast tissue, adipose tissue, and ducts with typical morphology.

## DISCUSSION

### Nutritional and functional potential of purple sweet potato

**Figure 3:**
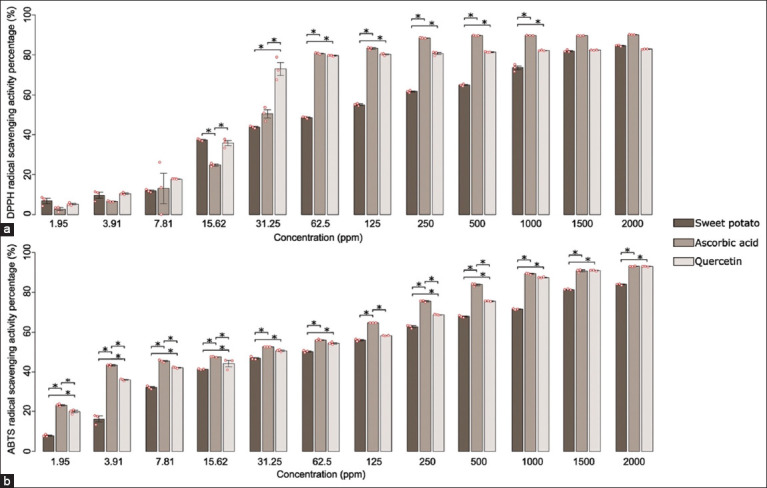
Determination of the antioxidant activity of purple sweet potatoes (*Ipomoea batatas*) using (a) the 2,2-diphenyl-1-picrylhydrazyl-hydrate and (b) 2,2’-Azino-bis(3-ethylbenzothiazoline-6-sulfonic acid) assay. All data are presented as mean ± standard error of the mean, n = 3 per group; analysis of variance, *post hoc* Tukey’s honestly significant difference test, (*) statistically significant difference between groups, p < 0.05.

Purple sweet potato (*I. batatas*) is a highly nutritious food source and a promising raw material for the development of functional foods [30]. It is recognized for its capacity to reduce oxidative damage and inflammation [31]. Although its antioxidant activity has been evaluated in malondialdehyde based assays, it has not been directly compared with established antioxidant agents such as quercetin or ascorbic acid. In the DPPH assay, the in vitro antioxidant activity of *I. batatas* was found to be comparable to quercetin at a concentration of 1500 ppm and statistically similar to ascorbic acid at the same concentration (p < 0.05), and a finding of significance considering the extract was derived from edible peels (Figure 3).

### Bioactive compounds and antioxidant mechanisms

Purple sweet potato is classified as a medicinal food due to its potent antioxidant properties, attributed primarily to its high content of anthocyanins, especially peonidin and cyanidin and their acylated derivatives [18, 32, 33]. In addition, it contains phenolic compounds such as 4-O-caffeoylquinic acid, 1,3-di-O-caffeoylquinic acid, and 3,5-di-O-caffeoylquinic acid [34], all of which have demonstrated anticancer activity in colon, gastric, and breast cancer models [33].

### DMBA-induced carcinogenesis model

DMBA is a well-established carcinogen used to investigate mammary gland tumorigenesis in rats, a species particularly susceptible to neoplastic transformation [35]. The highest sensitivity occurs when DMBA is administered between 45 and 60 days of age, coinciding with the onset of sexual maturity, marked by active mammary organogenesis and a high proliferation rate of type 1 and 2 lobules [36]. In this study, a single subcutaneous dose of DMBA (20 mg)-induced mammary tumor formation in female rats within 88–101 days. Histopathological analysis confirmed the presence of ductal carcinoma in the control group (Figure 2), consistent with the predominant type of breast cancer in women, as reported by the American Cancer Society, which states that most breast cancers originate in the ductal epithelium [37]. DMBA specifically targets the terminal ducts, inducing epithelial hyperplasia and promoting ductal carcinogenesis [38]. The mechanism of DMBA-induced carcinogenesis involves metabolic activation within the mammary gland, leading to the formation of the ultimate carcinogen DMBA-3,4-diol-1,2-epoxide, which promotes oxidative stress, DNA adduct formation, and mutations in terminal bud cells, ultimately contributing to malignant transformation [39, 40].

### Histological evidence of antitumor activity of *I. batatas*

Experimental groups treated with *I. batatas* extract demonstrated notable histological improvements in breast tissue, including well-differentiated structures. Inflammatory regions with intravascular infiltration were present, indicating a reduced degree of tumor severity compared to the DMBA control group, which exhibited extensive necrotic tissue [41]. These findings suggest that *I. batatas* exerts an antitumor effect in a dose-dependent manner (Figure 2). The observed protective effect is likely attributable to its high anthocyanin content, which is known to modulate oxidative stress, suppress inflammation, and promote apoptosis. These actions support the emerging role of anthocyanins as nutraceuticals in cancer prevention. In addition, this protection is crucial in maintaining tissue integrity and counteracting the oxidative damage induced by DMBA metabolism, which generates reactive oxygen species that contribute to lipid peroxidation, antioxidant depletion, and subsequent tissue injury – a hallmark of carcinogenesis [42, 43].

### Anthocyanins and molecular pathways in cancer prevention

The primary anthocyanins in purple sweet potato are peonidin 3-sophoroside-5-glucoside and cyanidin 3-glucoside, which exist in mono- or diacylated forms with caffeic, ferulic, and p-hydroxybenzoic acids. These acylated anthocyanins constitute more than 98% of the TAC in the purple variety [44, 45]. Anthocyanins are known to enhance cellular antioxidant and anti-inflammatory defenses and activate enzymes that neutralize carcinogens. They also inhibit cancer cell growth and dissemination [45, 46]. Mechanistically, anthocyanins interfere with critical signaling pathways, including AMPK, PI3K/AKT/mTOR, and JAK-STAT. Of these, the JAK-STAT pathway – particularly the inhibition of STAT3 – has been most extensively studied. STAT3 is heavily implicated in tumor progression, with aberrant activation observed in over 70% of human cancers. Furthermore, anthocyanins are capable of modulating both apoptotic and autophagic pathways, the latter acting as an alternative route for programmed cell death when apoptosis is compromised [47].

### Cyanidin: A key anticancer metabolite

Cyanidin, one of the major anthocyanins in *I. batatas* [48], is likely a key contributor to the extract’s anticancer effects. It has been shown to inhibit the JAK-STAT and vascular endothelial growth factor pathways and reduce STAT3 expression at both the mRNA and protein levels. Its chemopreventive action involves the regulation of cellular survival, proliferation, inflammation, and apoptosis [49].

### Future perspectives

Further research should aim to elucidate the molecular mechanisms by which anthocyanins from purple sweet potato exert anticancer effects [50], with a particular focus on their interactions with signaling pathways such as JAK-STAT, PI3K/AKT/mTOR, and apoptotic regulators [51]. In addition, well-designed clinical trials are necessary to confirm the therapeutic potential of these compounds in the treatment of human breast cancer.

## CONCLUSION

This study demonstrated that the ethanolic extract of purple sweet potato (*I. batatas* L.) peels exerts a significant chemopreventive effect against DMBA-induced breast carcinogenesis in rats. The extract, particularly at a dose of 600 mg/kg, significantly delayed tumor onset (101 days vs. 88 days in the DMBA control group), reduced tumor volume (2.26 cm^3^ vs. 15.21 cm^3^; p < 0.05), and improved histopathological profiles of mammary tissue. These effects are attributed primarily to the high anthocyanin content (138.92 ± 0.58 mg/100 g dry extract), with potent antioxidant activity comparable to quercetin and ascorbic acid, as evidenced by DPPH assay results.

The principal strength of this study lies in its integrative approach combining phytochemical quantification, antioxidant assessment, tumor monitoring, and histological analysis, all within a well-characterized DMBA-induced carcinogenesis model. In addition, the use of varying doses of the extract provided valuable insights into dose-dependent effects, supporting the therapeutic relevance of purple sweet potato anthocyanins.

Nevertheless, the study has several limitations. First, it was conducted exclusively in a rodent model, which may not fully replicate the complexity of human breast cancer. Second, while the extract’s anthocyanin content and antioxidant capacity were quantified, specific molecular targets and pathways (e.g., JAK-STAT3, PI3K/AKT/mTOR) were inferred but not directly examined through molecular or proteomic analyses. Third, the study did not include long-term follow-up or survival analyses, which are critical for assessing sustained chemopreventive effects.

## AUTHORS’ CONTRIBUTIONS

CRSC, JHV, and WEJC: Collected the plant species and drafted the manuscript. VEVLT and WASG: Preparation of extract and determination of antioxidant activity. CLAV and JLCR: Maintenance of animals during investigation and administration of treatments. AACP: Histopathological analysis. NBM and JCRB: Quantification of total anthocyanin. OHC and JACC: Statistical analysis and image preparation. All authors have read, reviewed, and approved the final version of the manuscript.
